# Immunomodulative Effects of *Chamaecyparis obtusa* Essential Oil in Mouse Model of Allergic Rhinitis

**DOI:** 10.3390/molecules25194517

**Published:** 2020-10-02

**Authors:** Seung-Heon Shin, Mi-Kyung Ye, Dong-Won Lee, Mi-Hyun Che

**Affiliations:** Department of Otorhinolaryngology-Head and Neck Surgery, School of Medicine, Catholic University of Daegu, Daegu 42472, Korea; miky@cu.ac.kr (M.-K.Y.); neck@cu.ac.kr (D.-W.L.); leonen@hanmail.net (M.-H.C.)

**Keywords:** allergic rhinitis, essential oil, *Chamaecyparis obtusa*, cytokine, transcription factor

## Abstract

The present study aims to investigate the immunomodulatory effects of essential oil from *Chamaecyparis obtusa* (EOCO) in an ovalbumin (OVA)-induced allergic rhinitis (AR) mouse model. BALB/c mice were intraperitoneally sensitized and stimulated with OVA. From day 22 to 35, 0.01% and 0.1% ECOC was intranasally administered 1 h before OVA stimulation. Nasal symptoms, as well as serum total and OVA-specific immunoglobulin (Ig) E levels, were measured. Interleukin (IL)-4, IL-10, interferon (IFN)-γ, and tumor necrosis factor (TNF)-α levels in nasal lavage fluid (NLF) and their production by activated splenocytes were measured. Histological changes in the sinonasal mucosa were evaluated through hematoxylin and eosin and periodic acid-Schiff staining procedure. Th cytokines and their transcription factor mRNA expressions were determined using reverse-transcription polymerase chain reaction. Intranasal EOCO administration significantly suppressed allergic symptoms, OVA-specific IgE level, sinonasal mucosal inflammatory cell infiltration, and mucus-producing periodic acid-Schiff (PAS) positive cell count. EOCO also significantly inhibited IL-4, IL-10, and TNF-α levels in NLF and activated splenocytes. Th2 and Treg related cytokines and their transcription factors in sinonasal mucosa were significantly suppressed through intransal EOCO instillation. In conclusion, repetitive EOCO intranasal instillation showed anti-inflammatory and anti-allergic effects by suppressing nasal symptoms and inhibiting the production and expression of inflammatory mediators in the OVA-induced AR mouse model.

## 1. Introduction

Allergic rhinitis (AR) is the inflammation of the nasal mucosa induced by a specific immunoglobulin E (IgE)-mediated hypersensitivity reaction against inhaled allergens, which involves Th2 immune responses. Immunologic interactions between epithelial cells, lymphocytes, mast cells, interleukin (IL)-4, IL-5, IL-13, and thymic stromal lymphopoietin (TSLP) are involved in AR [1]. A large number of pharmacologic and non-pharmacologic agents have been used to alleviate the symptoms and improve the quality of life in AR patients. However, most AR treatment modalities are focused on symptomatic control with palliative effects, and allergic immunotherapy is the only potential curative therapy that influences the immunopathologic mechanism in developing nasal mucosal hypersensitivity [2].

Essential oils are volatile compounds produced by plants as a protective mechanism against harmful insects and microorganisms. Several essential oils contain active substances that exert anti-fungal, anti-oxidative, and anti-gastropathic activity [3,4]. Essential oil extracted from *Chamaecyparis obtusa* (EOCO) consists of several monoterpenes, sesquiterpene hydrocarbons, oxygenated sesquiterpenes, and several other substances [5,6]. EOCO is used in soap, toothpaste, and aromatic agents with anti-bacterial, anti-atopic, and anti-inflammatory effects [5,7]. Active components from *Chamaecyparis obtusa* were found to inhibit lipopolysaccharide-induced nitric oxide, prostaglandins, and inflammatory cytokine production, thereby suppressing inflammatory cell infiltration and inflammatory mediator production, as observed through in vivo and in vitro studies [3,4]. EOCO can be a promising candidate for new pharmacologic agents.

Although the biological activities of EOCO are not fully understood, it has immune modulatory properties that influence both Th1 and Th2 immune responses [8]. The effects of EOCO in AR have yet to be investigated. In the present study, we used an AR mouse model to evaluate EOCO′s anti-allergic effect and its mechanism of action.

## 2. Results

### 2.1. Effects of EOCO on Allergic Behaviors

**Figure 1:**
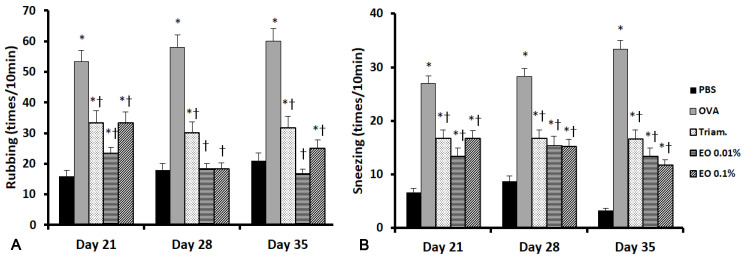
Effects of essential oil from *Chamaecyparis obtusa* (EOCO) in the allergic rhinitis mouse model (n = 8 mice per group). The frequency of nasal rubbing (**A**) and sneezing (**B**) counts was significantly decreased with intranasal instillation of triamcinolone (Triam.) and EOCO. PBS, phosphate-buffered saline; OVA, ovalbumin; * (*p* < 0.05) versus the PBS group; † (*p* < 0.05) versus the OVA group.

The effects of EOCO intranasal instillation on the frequency of nasal rubbing and sneezing after ovalbumin (OVA) stimulation at day 21, 28, and 35 were evaluated. OVA-stimulated mice sneezed significantly more (27.3 ± 8.1, 28.3 ± 7.4, and 33.3 ± 12.7 at days 21, 28, and 35, respectively) than the PBS-treated negative control mice (6.6 ± 2.7, 8.7 ± 2.3, and 3.3 ± 1.8 at days 21, 28, and 35, respectively). When mice were intranasally treated with triamcinolone (16.7 ± 3.5, 16.7 ± 6.8, and 16.6 ± 4.7 at days 21, 28, and 35, respectively) and EOCO (13.3 ± 5.7, 15.3 ± 4.5, and 13.3 ± 4.2 with 0.01% EOCO and 16.7 ± 6.8, 15.2 ± 7.6, and 11.7 ± 4.5 with 0.1% EOCO at days 21, 28, and 35, respectively), the frequency of sneezing was significantly decreased. The increased frequency of nasal rubbing in OVA-stimulated mice was also significantly inhibited by intranasal instillation of triamcinolone, as well as 0.01% and 0.1% EOCO (Figure 1).

### 2.2. Effects of EOCO on Total Serum IgE and OVA-Specific IgE Levels

**Figure 2:**
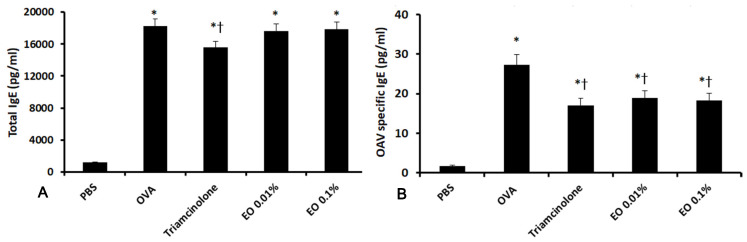
Effects of essential oil from *Chamaecyparis obtusa* (EOCO) on total serum IgE (**A**) and ovalbumin (OVA)-specific IgE (**B**) level (n = 8 mice per group). Triamcinolone suppressed both total serum IgE and OVA-specific IgE levels, whereas EOCO only suppressed OVA-specific IgE level. PBS, phosphate-buffered saline; OVA, ovalbumin; * (*p* < 0.05) versus the PBS group; † (*p* < 0.05) versus the OVA group.

The anti-allergic effects of EOCO on the immune response in AR mice were determined by measuring the total and OVA-specific IgE levels using enzyme-linked immunosorbent assay (ELISA) kits. The levels of total serum IgE and OVA-specific IgE after OVA stimulation (18,230.2 ± 2330.1 pg/mL and 21.3 ± 3.8 pg/mL, respectively) were markedly elevated, compared with those in the negative control (1202.3 ± 272.2 pg/mL and 1.8 ± 2.7 pg/mL, respectively) group. When mice were treated with triamcinolone, the total IgE and OVA-specific IgE were significantly inhibited. However, 0.01% and 0.1% EOCO only suppressed OVA-specific IgE, but not the total IgE (Figure 2).

### 2.3. Effects of EOCO on the Inflammatory Mediator Levels in Nasal Lavage Fluid (NLF)

IL-4, IL-10, IFN-γ, and TNF-α in NLF were measured. The levels of IL-4 (12.3 ± 5.2 pg/mL), IL-10 (9.2 ± 3.5 pg/mL), IFN-γ (5.1 ± 2.7 pg/mL), and TNF-α (7.0 ± 3.2 pg/mL) in the NLF were significantly greater in OVA-stimulated mice than those in the negative control mice (IL-4, 4.1 ± 2.5 pg/mL; IL-10, 4.6 ± 3.2 pg/mL; IFN-γ, 2.7 ± 2.1 pg/mL; and TNF-α, 5.3 ± 3.6 pg/mL). The levels of IL-4, IL-10, and TNF-α were significantly decreased by intranasal instillation of triamcinolone and EOCO, compared with those in OVA-sensitized mice. However, IFN-γ level in the NLF was only significantly influenced by intranasal instillation of 0.01% EOCO (2.5 ± 1.7 pg/mL), compared with OVA-stimulated mice (Table 1).

### 2.4. Effects of EOCO on the Inflammatory Mediator Production from Splenocytes

Isolated mouse splenocytes were stimulated with OVA for 72 h. The splenocytes of allergic mice produced larger amounts of IL-4 (15.0 ± 5.3 pg/mL), IL-10 (20.2 ± 7.5 pg/mL), IFN-γ (10.5 ± 5.4 pg/mL), and TNF-α (305.2 ± 86.7 pg/mL) after OVA stimulation. IL-4 production was significantly suppressed by triamcinolone (9.4 ± 3.6 pg/mL) and 0.1% EOCO (9.0 ± 2.7 pg/mL). IL-10 production was significantly suppressed by 0.01% (13.8 ± 5.2 pg/mL) and 0.1% (15.5 ± 6.3 pg/mL) EOCO, and TNF-α was only significantly inhibited by 0.1% EOCO (237.8 ± 112.3 pg/mL). However, the increased production of IFN-γ by OVA stimulation was not inhibited by the triamcinolone and EOCO treatments (Table 2).

### 2.5. Effects of EOCO on the Expression of Inflammatory Mediators and Transcription Factors in the Sinonasal Mucosa

**Figure 3:**
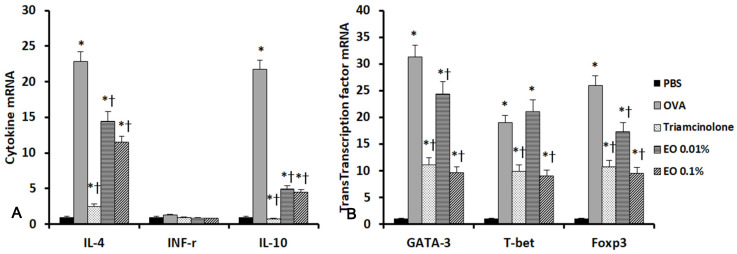
Effects of essential oil from *Chamaecyparis obtusa* (EOCO) on the expression of inflammatory mediator (**A**) and transcription factor mRNAs (**B**) in the sinonasal mucosa (n = 4 mice per group). IL-4 and IL-10, as well as their transcription factor mRNAs’ expressions, were significantly inhibited by intrannasal EOCO instillation. PBS, phosphate-buffered saline; OVA, ovalbumin; * *p* < 0.05 versus the PBS group; † *p* < 0.05 versus the OVA group.

We performed real-time RT-PCR to determine the influence of EOCO intranasal instillation on the expression of cytokines and their transcription factor mRNAs in the sinonasal mucosa. IL-4 and GATA-3, IL-10 and Foxp3, and T-bet mRNA expressions were significantly increased in allergic mice. IL-4 and GATA-3 expressions, as well as IL-10 and Foxp3 mRNA expressions, were significantly inhibited by intranasal instillation of triamcinolone and EOCO. Although IFN-γ mRNA expression was not significantly different among the five groups, T-bet mRNA expressions were significantly inhibited by triamcinolone and 0.1% EOCO (Figure 3).

### 2.6. Effects of EOCO on Sinonasal Mucosal Inflammation

**Figure 4:**
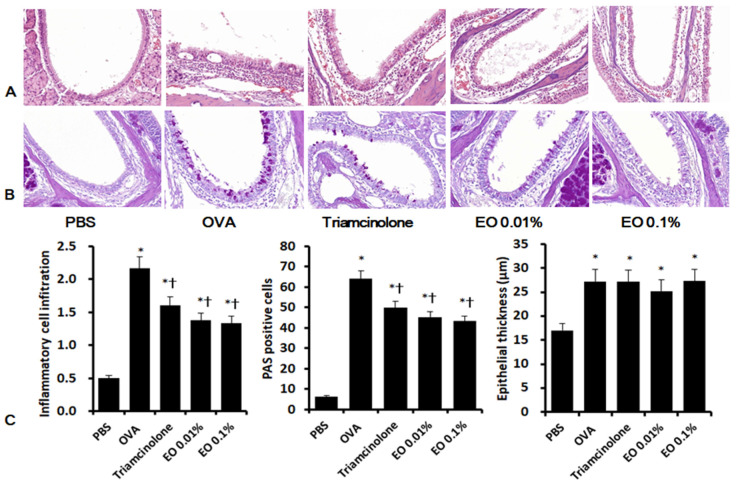
Effects of essential oil from *Chamaecyparis obtusa* (EOCO) on the histologic characteristics of sinonasal mucosa (n = 4 mice pergroup). Inflammatory cell infiltration and periodic acid-Schiff (PAS)-positive cells were significantly decreased by intrannasal EOCO instillation (**C**). **A**, representative photographs of hematoxylin and eosin (H&E) stained tissues; **B**, representative photographs of PAS stained tissues; PBS, phosphate-buffered saline; OVA, ovalbumin; * *p* < 0.05 versus the PBS group; † *p* < 0.05 versus the OVA group.

Intranasal OVA stimulation (2.2 ± 0.6) significantly increased inflammatory cell infiltration compared with untreated negative control mice (0.5 ± 0.4). Intranasal instillation of triamcinolone (1.6 ± 0.5) and EOCO (1.4 ± 0.8 for 0.01% EOCO and 1.3 ± 0.8 for 0.1% EOCO) significantly suppressed inflammatory cell infiltration, compared with OVA-stimulated mice. Eosinophil infiltration was also significantly inhibited by intranasal instillation of triamcinolone (13.8 ± 5.2) and EOCO (29.5 ± 13.7 for 0.01% EOCO and 25.0 ± 9.4 for 0.1% EOCO), compared with untreated mice (44.0 ± 15.6). Periodic acid–Schiff (PAS)-positive goblet cells in the sinonasal mucosa showed a significant increases in OVA-stimulated mice (64.0 ± 16.4), compared with the control mice (6.4 ± 2.8). Intranasal instillation of triamcinolone (49.9 ± 21.7) and EOCO (45.4 ± 16.2 for 0.01% EOCO and 43.3 ± 19.4 for 0.1% EOCO) significantly influenced the number of PAS-positive cells. However, the increased thickness of epithelial cells in the sinonasal mucosa in OVA-stimulated mice was not influenced by intranasal instillation of triamcinolone or EOCO (Figure 4).

## 3. Discussion

AR is an immediate type I hypersensitivity reaction, characterized by the recruitment of inflammatory cells, increased pro-inflammatory mediators, and Th2 cytokines in the sinonasal mucosa. Inflammatory symptoms are mainly triggered by inflammatory mediators, such as histamine; IL-4; IL-6; and several other inflammatory mediators produced by inflammatory cells, such as mast cells, eosinophils, basophils, and T lymphocytes [9]. Several pharmacologic and immunologic agents are used to control allergic symptoms [10]. Additional new promising pharmacologic and immunologic approaches will certainly change the pattern of the treatment modalities for AR, although most of the therapeutic agents used in treating AR are effective and safe. In the present study, EOCO showed anti-inflammatory and anti-allergic effects in an AR mouse model.

*C. obtusa* is a tropical tree species commonly found in Japan and the southern region of Korea. EOCO contains a large amount of phytoncides, a natural antibiotic volatile compound that protect plants from harmful insects, animals, or microorganisms. EOCO contains several types of monoterpenes, sesquiterpene hydrocarbons, oxygenated sesquiterpenes, and several others substances. EOCO has been reported to reduce the production of prostaglandin E2, TNF-α, and cyclo-oxygenase-2, with anti-microbial, anti-fungal, anti-oxidative, and anti-inflammatory properties [11,12,13]. We used microencapsulated essential oil for the present study, because essential oils are volatile and hydrophobic. This microencapsulation protects active compounds from environmental factors, such as oxygen, light, and moisture, and increases their solubility in water [8,14]. Stylene maleic anhydride polymer is a synthetic polymer that is used in microencapsule formation through interfacial polycondensation or complex coacervation.

The results of the present study showed the anti-inflammatory and anti-allergic effects of EOCO in an AR mouse model. Allergic symptoms mainly originated from inflammatory cells in response to inflammatory cells-derived mediators [15]. Topical blockage of TNF-α reduced pathologic allergic reaction in the sinonasal mucosa of AR mice [16]. Intranasal instillation of EOCO significantly suppressed IL-4, IL-10, and TNF-α levels in the NLF, with decreased AR symptoms. Although IL-4 and IL-10 mRNA expressions were more strongly suppressed by triamcinolone, 0.01% and 0.1% of EOCO also significantly suppressed these mRNAs’ expressions. Triamcinolone and EOCO suppressed IL-4- and IL-10-related transcription factors. EOCO suppressed the expression of T-bet, an IFN-γ related transcription factor. However, EOCO did not influence IFN-γ mRNA expression. This discrepancy may be related to the low IFN-γ mRNA expression level in the AR mouse model. The IFN-γ level in the NLF tended to be decreased by EOCO, although we did not measure IFN-γ protein level in the sinonasal mucosa. Intranasal EOCO instillation influenced Th2 and Treg immune responses in the AR mouse model and the anti-inflammatory potency was very similar to triamcinolone. In the present study, however, we cannot draw a direct conclusion of whether or not EOCO could influence Th1 immune response.

Serum total and OVA-specific IgE levels were significantly increased in OVA-stimulated mice. As an exogenous antigen, OVA stimulates B cells to produce IgE and activate mast cells [17]. Intranasal instillation of EOCO suppressed the serum OVA-specific IgE level, but did not influence the total serum IgE level. However, OVA-specific IgE levels in EOCO-treated mice were much higher than those of negative control mice. The IL-4, IL-10, and TNF-α levels in the NLF were significantly low in both 0.01% and 0.1% EOCO-treated mice compared with those in OVA-stimulated mice. When splenocytes were stimulated with OVA, IL-4, IL-10, and TNF-α production significantly increased in OVA-stimulated mice, and the production of these inflammatory mediators was decreased in the splenocytes of mice that were intranasally treated with 0.1% EOCO. Intranasal EOCO instillation may more strongly modulate local inflammatory responses than systemic inflammatory responses, although we cannot draw a conclusion from the results of the present study. If we measured OVA-specific IgE in the NLF, it might appear to be as low as that in negative control mice. These findings indicate that intranasal EOCO instillations may effectively inhibit local inflammation with only few systemic effects using higher concentrations of EOCO.

Inflammatory cell infiltration and mucus hypersecretion are pathognomonic characteristics of airway inflammatory diseases. Tissue eosinophilia plays a crucial role in AR. The OVA-induced allergic mouse model showed increased inflammatory cell infiltration with tissue eosinophilia. Inflammatory cell infiltration was downregulated by EOCO, and the increased eosinophil count was significantly suppressed through intranasal instillation of triamcinolone (68.6%) and EOCO (33.0% for 0.01% EOCO and 43.2% for 0.1% EOCO). Pro-inflammatory cytokines, including IL-6 and IL-8, have been known as major cytokines involved in mucus hypersecretion and AR pathogenesis [18]. Intranasal EOCO instillation suppressed mucus-producing cells in the sinonasal mucosa. On the basis of these results, EOCO inhibits mucus production by suppressing the production of pro-inflmmatory mediators in an AR mouse model.

## 4. Materials and Methods

### 4.1. Preparation of Microencapsulated EOCO

Qwell Inc. (Seoul, Korea) kindly provided microencapsulated EOCO. Essential oil was extracted from leaves of *C. obtusa* collected in Masan, Kyunsangnamdo, Korea. EOCO was produced through steam distillation of leaves according to previously described methods [9]. The collected essential oil was stored at room temperature in nitrogen tanks for 1 year to stabilize its components and be suitable for daily products. The nitrogen tank prevents the evaporation and composition change of EOCO. The composition of EOCO was determined using a gas chromatography mass spectrometry analysis (Agilent Tech., Santa Clara, CA, USA). Essential oil contained more than 20 components and Table 3 shows the main chemical composition of ECOC used in this study. For microencapsulation, EOCO was mixed with a water-soluble stylene maleic anhydride polymer, and melanin pre-condensate was added to the mixture, resulting in the formation of microcapsules consisting of a spherical inner core and an outer shell.

### 4.2. Preparation of OVA-Induced AR Mouse Model

Pathogen-free female BALB/c mice (aged 6–8 weeks) were obtained from Hyosung Science Inc. (Daegu, Korea). They were kept under specific pathogen-free conditions. This study was conducted in accordance with the guidelines of the National Institute of Health and approved by the Institutional Review Board of Animal Experiments of Daegu Catholic University Medical Center.

Mice were sensitized through intraperitoneal injection with 75 µg of OVA in 200 µL of phosphate-buffered saline (PBS) containing 2 mg of aluminum hydroxide (Sigma–Aldrich, St. Louis, MO, USA) on days 0, 7, 14, and 21. On days 22–35 after initial sensitization, mice were stimulated through bilateral intranasal instillation with 500 μg of OVA in 20 μL of PBS. The mice were randomly divided into five groups (n = 8). The negative control mice were stimulated with PBS instead of OVA (group I). To determine the anti-allergic and anti-inflammatory effect of EOCO, 20 μL of 0.01% (group IV) and 0.1% (group V) EOCO dissolved in PBS was intranasally instilled using a micropipette 1 h before each stimulation on days 22–35. The control group was treated with PBS (group II) or 0.5 mg/kg of triamcinolone (group III).

### 4.3. Evaluation of Nasal Symptoms

The frequency of nasal rubbing and sneezing was recorded during a period of 15 min after stimulation on days 21, 28, and 35, and the results of each group were compared.

### 4.4. Evaluation of Nasal Lavage Fluid (NLF)

NLF was collected 24 h after the last intranasal stimulation at day 35. A 21-gauge catheter was inserted through a partial tracheal resection site in the direction of the upper airway and into the nasopharynx. The nasal cavity was gently perfused with 1 mL of cold PBS. The collected NLF was centrifuged at 2000 rpm for 7 min at 4 ℃. The amount of interleukin (IL)-4, IL-10, interferon (IFN)-γ, and tumor necrosis factor (TNF)-α in the NLF was determined using enzyme-linked immunosorbent assay (ELISA) kits (R&D Systems, Minneapolis, MN, USA).

### 4.5. Measurement of Serum IgE

Blood samples were collected from the inferior vena cava 24 h after the last intranasal stimulation. Serum was obtained by centrifugation, and total and OVA-specific IgE were measured using ELISA kits (Pharmingen, San Diego, CA, USA).

### 4.6. Measurement of Cytokines and Transcription Factor mRNAs in the Sinonasal Mucosa

Total RNA was extracted from the sinonasal mucosa using Trizol reagent (Invitrogen, Carlsbad, CA, USA). RNA purity and concentration were measured through spectrophotometery (Beckman, Mountain View, CA, USA). Complementary DNA was made from 1 µg of RNA through reverse-transcription polymerase chain reaction (RT-PCR) amplification with a thermal cycler (PerkinElmer Corp., Norwalk, CT, USA). From the amplified cDNA, the quantitative PCR was performed using a SYBR Green PCR core kit (PE Applied Biosystems, Foster City, CA, USA). The primer sequences and amplification products were as follows: IL-4 sense 5′- CAATTGCAATGCCATCTACAGGAC-3′ and antisense 5′-TTTTGGTATCGGGGAGGCTG-3′ (104 bp); IL-10 sense 5′-GCCAGAGCCACATGCTCCTA-3′ and antisense 5′-GATAAGGCTTGGCAACCCAAGTAA-3′ (145 bp); IFN-γ sense 5′-CGGCACAGTCATTGAAAGCCTA-3′ and antisense 5′-GTTGCTGATGGCCTGATTGTC-3′ (199 bp); T-bet sense 5′-GCCAGGGAACCGCTTATA-3′ and antisense 5′-CCTTGTTGTTGGTGAGCTTTA-3′ (104 bp); GATA-3 sense 5′-TACCACCTATCCGCCCTATG-3′ and antisense 5′-GCCTCGACTTACATCCGAAC-3′ (101 bp); Foxp3 sense 5′-CACCTATGCCACCCTTATCCG-3′ and antisense 5′-CATGCGAGTAAACCAATGGTAGA-3′ (91 bp); and to *β-actin sense* 5′-GCAGAAGGAGATTACTGCTCT-3′ and antisense 5′-GCTGATCCACATCTGCTGGAA-3′ (136 bp). Initial denaturation was performed at 95 °C for 2 min, followed by 40 cycles consisting of denaturation at 94 °C for 10 s, annealing at 60 °C for 10 s, and elongation at 72 °C for 45 s. All samples were amplified in triplicate. The expression levels of the aformentioned mRNA were normalized to the median value for *β-actin* and expression levels were measured using the cycle threshold method (2^−ΔΔ^CT).

### 4.7. Measurement of Cytokines from Splenocytes with OVA

Splenocytes were isolated from spleen tissues by grounding and separating the tissue into single cells using a 70 µm cell strainer. Red blood cells (RBCs) were removed using an RBC lysis buffer (BioLegend, San Diego, CA, USA). The splenocytes were incubated in Roswell Park Memorial Institute 1640 medium supplemented with 10% fetal bovine serum, 100 U/mL of penicillin, and 100 µg/mL of streptomycin (Gibco, Grand Island, NY, USA). After stimulation with 100 µg/mL of OVA for 72 h, the supernatant was collected, and IL-4, IL-10, IFN-γ, and TNF-α levels were measured using ELISA kits (R&D Systems).

### 4.8. Histologic Evaluation of Sinonasal Mucosa

Mice were anesthetized with 1% pentobarbital sodium and painlessly sacrificed 24 h after the last intranasal stimulation at day 36. The specimens were decalcified in ethylenediaminetetraacetic acid and then immersed in 4% paraformaldehyde for 24 h. The paraffin embedded tissue was anteroposterioly sectioned at a 5 μm thickness, and three anatomically similar sections were chosen from each mouse for analysis [19].

Inflammatory cell infiltration and epithelial thickness were quantified in hematoxylin and eosin (H&E) stained sections. Eosinophil infiltration was calculated as the average number of cells in five high power fields. The degree of submucosal inflammatory cell infiltration was quantified into four categories as follows: 0, none; 1, mild occasional scattered inflammatory cells; 2, moderate; and 3, severe diffuse infiltration of inflammatory cells. Goblet cell hyperplasia was determined by periodic acid-Schiff (PAS) staining, and the average number of goblet cells was counted using an eyepiece reticle at ×200 magnification. Epithelial thickness was directly measured at ×400 magnification through a video camera (Olympus Optical Co. Ltd., Tokyo, Japan) and analyzed using the DP Controller software (ver. 2.2.1.227). All tissue sections were blindly examined in terms of tissue origin, and the mean counts were determined at three different mucosal areas for each of the three sections per mouse.

### 4.9. Statistical Analysis

All measured parameters are expressed as mean ± standard deviation of eight representative independent experiments for each group. The comparison between two groups was analyzed using Student’s t-test and data among several groups were analyzed by one-way analysis of variance (ANOVA) followed by Tukey’s test for normally distributed data. The Mann–Whitney *U* test and the Kruskal–Wallis test with post-hoc Bonferroni–Dunn test were performed for non-normally distributed data using the SPSS ver. 21 software (IBM Corp., Armonk, NY, USA). A *p-*value of <0.05 was considered statistically significant.

## 5. Conclusions

Repetitive intranasal EOCO instillation resulted in anti-inflammatory and anti-allergic effects, with the suppression of nasal symptoms by inhibiting the production and expression of inflammatory mediators in the OVA-induced AR mouse model. Intranasal EOCO instillation more strongly suppressed local inflammatory responses and elicited weaker systemic effects. The anti-inflammatory and anti-allergic effect of EOCO was very similar with triamcinolone, which is the representative glucocorticosteroid. These findings suggest that EOCO could be a good candidate for the production of a local anti-inflammatory and anti-allergic agent with low systemic side effects.

## Figures and Tables

**Table 1:** Effects of essential oil from *Chamaecyparis obtusa* (EOCO) on the level of inflammatory mediators in the nasal lavage fluid (NLF) (n = 8 mice per group).

	NC	OVA	Triamcinolone	EOCO 0.01%	EOCO 0.1%
IL-4 (pg/mL)	4.08 ± 2.46	12.28 ± 5.24 ^a^	8.22 ± 2.23 ^a,b^	7.35 ± 1.45 ^a,b^	5.58 ± 1.65 ^b^
IFN-r (pg/mL)	2.65 ± 2.13	5.06 ± 2.65 ^a^	4.41 ± 0.68	2.50 ± 1.65 ^b^	4.55 ± 1.45
IL-10 (pg/mL)	4.58 ± 3.21	9.16 ± 3.48 ^a^	2.86 ± 0.77 ^b^	3.33 ± 1.36 ^b^	6.58 ± 1.82b ^a^
TNF-a (pg/mL)	5.29 ± 3.57	7.02 ± 3.22	5.81 ± 1.82 ^b^	5.40 ± 1.86 ^b^	5.35 ± 0.60 ^b^

NC, negative control; OVA, ovalbumin; ^a^ *p* < 0.05, significantly different from the NC group; ^b^ *p* < 0.05, significantly different from the OVA group.

**Table 2:** Effects of essential oil from *Chamaecyparis obtusa* (EOCO) on the production of inflammatory mediators by splenocytes after stimulation with 100 µg/mL of ovalbumin (OVA) (n = 8 mice per group).

	NC	OVA	Triamcinolone	EOCO 0.01%	EOCO 0.1%
IL-4	5.47 ± 1.45	14.95 ±5.34 ^a^	9.43 ± 3.56 ^a,b^	15.55 ± 4.74 ^a^	9.02 ± 2.72 ^a,b^
IFN-r	6.85 ± 2.52	10.48 ± 5.37 ^a^	8.70 ± 5.12 ^a^	9.16 ± 2.86 ^a^	8.39 ± 2.94 ^a^
IL-10	6.86 ± 1.60	20.20 ± 7.47 ^a^	25.93 ± 7.71 ^a^	13.84 ± 5.21 ^a,b^	15.52 ± 6.33 ^a,b^
TNF-a	134.69 ± 57.45	305.18 ± 86.73 ^a^	302.52 ± 60.24 ^a^	288.18 ± 78.54 ^a^	237.81 ± 112.34 ^a,b^

NC, negative control; OVA, ovalbumin; ^a^ *p* < 0.05, significantly different from the NC group; ^b^ *p* < 0.05, significantly different from the OVA group.

**Table 3:** Main components of essential oil from *Chamaecyparis obtusa*.

Compound	Kovatz Index	Retention Time (min)	Peak Area (%)
α-Pinene	928	8.75	5.96
Sabinene	966	10.96	16.72
Myrcene	989	11.36	19.45
α-Terpinene	1017	12.69	3.05
γ-Terpinene	1044	13.54	4.75
Limonene	1028	13.76	1.82
Terepineol	1095	15.21	1.26
Terpinene-4-ol	1162	16.23	2.82
Bornyl acetate	1265	27.53	9.46
α -Terpinyl acetate	1349	28.64	15.69
